# Chronic Low-Grade Inflammation: A Possible Link Between COVID-19 and New-Onset Atrial Fibrillation

**DOI:** 10.3390/jcm15051750

**Published:** 2026-02-25

**Authors:** Ciprian Ilie Roșca, Daniel Florin Lighezan, Daniel-Dumitru Nișulescu, Nilima Rajpal Kundnani, Romina Georgiana Bita, Ariana Violeta Nicoras, Christian Banciu, Andreea Munteanu

**Affiliations:** 1 Department V, Internal Medicine I—Discipline of Medical Semiology I, Center of Advanced Research in Cardiology and Hemostasology, “Victor Babeș” University of Medicine and Pharmacy, Eftimie Murgu Sq. no. 2, 300041 Timișoara, Romania; rosca.ciprian@umft.ro (C.I.R.); dlighezan@umft.ro (D.F.L.); 2 Department of Histology, Faculty of Medicine, “Vasile Goldis” Western University of Arad, 310025 Arad, Romania; 3 Department of Cardiology—Ambulatory Internal Medicine, “Victor Babeș” University of Medicine and Pharmacy, Eftimie Murgu Sq. no. 2, 300041 Timișoara, Romania; 4 2nd Department, Radiology and Medical Imaging, General and Dento-Maxillary Imaging, Dental Medicine Faculty, “Victor Babeș” University of Medicine and Pharmacy, 300041 Timisoara, Romania; romina.bita@umft.ro; 5 General Medicine Faculty, “Victor Babeș” University of Medicine and Pharmacy, Eftimie Murgu Sq. no. 2, 300041 Timișoara, Romania; ariana.nicoras@umft.ro (A.V.N.); munteanu.andreea@umft.ro (A.M.); 6 Department V, Internal Medicine I—Discipline of Internal Medicine IV, Center of Advanced Research in Cardiology and Hemostasology, “Victor Babeș” University of Medicine and Pharmacy, Eftimie Murgu Sq. no. 2, 300041 Timisoara, Romania; banciu.christian@umft.ro

**Keywords:** COVID-19, SARS-CoV-2, endothelial dysfunction, flow-mediated dilation (FMD), inflammation, interleukin-6 (IL-6), neutrophil-to-lymphocyte ratio (NLR), atrial fibrillation, Holter monitoring, thrombosis/coagulation markers

## Abstract

**Background:** Persistent inflammation and endothelial dysfunction have been proposed as key mechanisms of post-COVID cardiovascular sequelae and may contribute to atrial fibrillation (AF). We examined whether inflammatory/prothrombotic biomarkers and endothelial function differ between post-COVID patients and controls, and whether baseline inflammation/endothelial dysfunction relates to AF burden at 12 months. **Methods**: In this single-center, retrospective observational study, 198 outpatients were enrolled: 99 post-COVID patients evaluated 3–6 months after documented SARS-CoV-2 infection (Group 1) and 99 age- and sex-matched controls without prior COVID-19 (Group 2). At baseline (t0), clinical characteristics, inflammatory/prothrombotic biomarkers, brachial artery flow-mediated dilation (FMD), and 24 h Holter ECG were assessed in both groups. Univariable linear regression tested associations between baseline variables and FMD in Group 1. At 12 months (t1), 24 h Holter ECG was repeated in both groups. Quartile analyses were performed according to baseline neutrophil-to-lymphocyte ratio (NLR) to explore AF distribution across inflammatory strata. **Results**: At baseline, post-COVID patients had higher inflammatory and prothrombotic markers than controls (ESR, CRP, fibrinogen, and D-dimer; all *p* < 0.0001) and markedly lower FMD (7.72 vs. 13.72; *p* < 0.0001). In Group 1, FMD was inversely associated with multiple inflammatory/prothrombotic markers (all *p* < 0.0001), with the strongest association for ESR (R^2^ = 0.6297). Holter-detected AF prevalence at baseline did not differ significantly between groups (25/99 [25.3%] vs. 18/99 [18.2%]). At 12 months, AF prevalence was numerically higher in the post-COVID group (32/99 [32.3%] vs. 21/99 [21.2%]); on two-sided testing, this difference was borderline (*p* = 0.047) and should be interpreted cautiously. Across increasing baseline NLR quartiles, AF prevalence increased stepwise in both groups (post-COVID: 2/25, 5/25, 10/24, 15/25; controls: 1/25, 3/25, 7/24, 10/25), consistent with the enrichment of AF in higher-inflammatory strata. **Conclusions**: Post-COVID patients exhibited a persistent inflammatory–prothrombotic profile and pronounced endothelial dysfunction at baseline. At 12 months, AF burden was numerically higher post-COVID, and AF clustered in strata characterized by higher baseline NLR and lower FMD, consistent with an inflammation–endothelial dysfunction axis associated with subsequent AF burden. Prospective studies with standardized rhythm monitoring and comprehensive multivariable adjustment are warranted.

## 1. Introduction

Since late 2019, the COVID-19 pandemic caused by SARS-CoV-2 has had a profound global health impact, with clinical manifestations ranging from asymptomatic infection to severe pneumonia and acute respiratory distress syndrome. Beyond the acute phase, accumulating evidence indicates that a substantial proportion of individuals experience persistent symptoms and multi-organ sequelae, including cardiovascular complications, which remain a major focus of ongoing research [1,2].

A key element in COVID-19 pathobiology is the interaction between SARS-CoV-2 and the angiotensin-converting enzyme 2 receptor (ACE2), which facilitates viral entry and is widely expressed in pulmonary, cardiac, vascular, renal, and intestinal tissues. Viral binding and subsequent ACE2 dysregulation can trigger a complex inflammatory cascade and endothelial activation, contributing to systemic inflammation, microvascular injury, and disturbed vascular homeostasis. These pathways are closely associated with the thrombo-inflammatory phenotype described in COVID-19 and are thought to underpin both acute cardiovascular events and longer-term vascular dysfunction [1,3,4,5].

Endothelial dysfunction has emerged as a central mechanism in post-COVID cardiovascular pathology. Inflammatory signaling, oxidative stress, and immune cell activation can impair endothelial nitric oxide bioavailability, disrupt the endothelial barrier, and promote procoagulant and prothrombotic states. Importantly, in some patients, these abnormalities may persist after clinical recovery, potentially sustaining microvascular dysfunction and contributing to long-term cardiovascular risk [5,6,7].

Atrial fibrillation (AF), one of the most common cardiac arrhythmias, has been increasingly reported during and after COVID-19. Inflammation is a recognized contributor to atrial electrical instability and remodeling, and biomarkers reflecting systemic inflammation and innate immune activation—such as interleukin-6 (IL-6) and the neutrophil-to-lymphocyte ratio (NLR)—have been associated with AF occurrence and adverse outcomes. Endothelial dysfunction may further amplify arrhythmogenic susceptibility through impaired vascular signaling, prothrombotic activation, and adverse interactions with comorbid conditions such as hypertension, dyslipidemia, obesity, and heart failure with preserved ejection fraction (HFpEF) [8].

Flow-mediated dilation (FMD) of the brachial artery is a widely used, noninvasive method to quantify endothelial function and to estimate vascular health. Reduced FMD has been associated with systemic inflammation and elevated cardiovascular risk, and may provide clinically relevant insight into post-infectious vascular impairment. However, the relationship between inflammatory/prothrombotic biomarkers, endothelial dysfunction measured by FMD, and subsequent AF burden in post-COVID patients—particularly assessed through rhythm monitoring—remains incompletely characterized in real-world cohorts [9,10,11].

Prior post-COVID studies have documented impaired FMD and persistent inflammatory activation, but most did not integrate endothelial function testing with longitudinal Holter-based atrial fibrillation ascertainment in a real-world outpatient setting. By combining inflammatory/prothrombotic profiling, standardized FMD assessment, and repeat 24 h Holter monitoring at 12 months, our study adds incremental evidence on how persistent post-COVID inflammation and endothelial dysfunction relate to subsequent AF burden.

Therefore, the present study aimed to investigate, in an age- and sex-matched outpatient cohort, (i) differences in inflammatory and prothrombotic markers and endothelial function between post-COVID patients and controls at baseline, (ii) the association between baseline inflammatory burden and FMD within the post-COVID group, and (iii) the relationship between baseline IL-6/NLR/FMD quartiles and atrial fibrillation burden assessed by 24 h Holter ECG at 12-month follow-up. We also considered the potential influence of cardiometabolic comorbidities on these relationships, given their known contribution to both endothelial dysfunction and AF risk [7,12].

## 2. Materials and Methods

### 2.1. Study Design and Setting

This was a single-center, retrospective observational study conducted at the Internal Medicine Clinic of the CFR Clinical Hospital, Timișoara, Romania. Consecutive eligible outpatients evaluated up to 1 November 2024 were screened. The study was performed in accordance with the Declaration of Helsinki and was approved by the local Ethics Committee. Data were extracted from medical records and processed in anonymized form.

### 2.2. Study Population and Group Allocation

A total of 198 adult participants were included and allocated 1:1 into two groups:

- Group 1 (Post-COVID, *n* = 99): patients with documented SARS-CoV-2 infection, clinically recovered, evaluated 3–6 months after infection.
- Group 2 (Controls, *n* = 99): patients without prior COVID-19 history, recruited from the same outpatient setting and matched to Group 1 by age and sex.

### 2.3. Inclusion and Exclusion Criteria

Inclusion criteria: age ≥ 18 years; availability of key baseline clinical data; completion of baseline endothelial function assessment and rhythm monitoring.

Exclusion criteria: known AF prior to baseline assessment; significant structural heart disease (moderate-to-severe valvular disease or known cardiomyopathy); prior myocardial infarction or coronary revascularization; active malignancy; chronic systemic inflammatory/rheumatologic disease; severe renal impairment (e.g., eGFR < 30 mL/min/1.73 m^2^); pregnancy; missing key baseline measurements.

Cardiometabolic conditions (hypertension, obesity, diabetes, dyslipidemia) and chronic therapies were not exclusion criteria; they were recorded and handled as potential confounders.

### 2.4. Study Timepoints and Outcomes

- Baseline (t0): performed at study entry (Group 1: 3–6 months post-infection; Group 2: baseline visit in the same calendar period).
- Follow-up (t1): approximately 12 months after t0.

Primary rhythm outcome: AF presence on 24 h Holter ECG at each timepoint. Because AF detection at each timepoint reflects the proportion of participants with AF at that assessment, outcomes are reported as AF prevalence at t0 and t1.

### 2.5. Clinical Data Collection and Definitions

Demographic and clinical variables were retrieved from outpatient charts and electronic records: age, sex, body mass index (BMI), smoking status, and comorbidities.

Definitions:

- Obesity: BMI ≥ 30 kg/m^2^.
- Hypertension: documented diagnosis and/or office blood pressure ≥ 140/90 mmHg and/or ongoing antihypertensive therapy.
- Dyslipidemia: documented diagnosis and/or lipid-lowering therapy and/or abnormal lipid profile based on laboratory reference ranges.
- HFpEF: documented diagnosis supported by available echocardiographic data and guideline-consistent criteria when present in records.

### 2.6. Laboratory Measurements

Baseline (t0) inflammatory and prothrombotic biomarkers were extracted from the hospital laboratory database, including erythrocyte sedimentation rate (ESR), fibrinogen, C-reactive protein (CRP), D-dimer, ferritin, interleukin-6 (IL-6), and lactate dehydrogenase (LDH). All assays were performed in the central hospital laboratory using routine standardized methods. Biomarkers were analyzed as continuous variables.

### 2.7. Assessment of Endothelial Function

Endothelial function was assessed at baseline (t0) using brachial artery flow-mediated dilation (FMD) with a Fujifilm Diagnostic Ultrasound System Arietta 65. Participants were examined supine after ≥10 min of rest in a quiet, temperature-controlled environment. The brachial artery was imaged longitudinally above the antecubital fossa. Baseline diameter (Di) was measured at end-diastole. A pneumatic cuff placed proximal to the measurement site was inflated to suprasystolic pressure (≥50 mmHg above systolic blood pressure) for 5 min, followed by rapid deflation. Post-deflation diameter (Df) was measured at a standardized timepoint after cuff release (e.g., 60 s). FMD was calculated as

FMD (%) = [(Df − Di)/Di] × 100

FMD was primarily analyzed as a continuous variable. All FMD measurements were performed according to a standardized protocol by trained operators using the same ultrasound system, minimizing inter-observer variability.

### 2.8. Rhythm Assessment and Definition of Atrial Fibrillation

AF was assessed by standard 12-lead ECG during clinical evaluation and by 24 h Holter ECG.

- Baseline Holter (t0): performed in both groups.
- Follow-up Holter (t1): repeated in both groups at ~12 months after t0; no loss to follow-up occurred (99/99 in each group).

AF was defined as an episode of atrial fibrillation lasting ≥30 s documented on ECG or Holter recording.

Reporting:

- AF prevalence at t0: proportion of participants with AF on baseline Holter, compared between groups.
- AF prevalence at t1: proportion of participants with AF on follow-up Holter, compared between groups.

### 2.9. Echocardiographic Data

Because this was a retrospective outpatient cohort, echocardiographic examinations were not performed under a single standardized protocol for all participants. Where available in the medical record, routine parameters of cardiac structure and function (e.g., LVEF and left atrial dimensions) were reviewed. However, as these measures were not uniformly available for all participants, they were not included as covariates in the primary adjusted models; instead, HFpEF diagnosis was used as a clinical proxy for relevant structural/functional vulnerability. Future prospective studies should incorporate systematic echocardiography and strain imaging to better account for myocardial injury and atrial remodeling.

### 2.10. Quartile Analysis

Participants were stratified into quartiles based on baseline NLR (t0) (Q1 lowest to Q4 highest). For each quartile, IL-6 and FMD were summarized descriptively, and AF prevalence at follow-up (t1) was reported as n/N (%) within each quartile for each group. The association between baseline NLR quartiles and AF at t1 was evaluated using Chi-square testing and a test for trend (Cochran–Armitage). In addition, we estimated regression-based odds ratios using logistic regression with NLR quartile as an ordinal predictor (per-quartile increase), fitted separately within each group. A pooled model including group, quartile, and a group × quartile interaction term was used to explore effect modification. Given the limited number of observations per quartile stratum, these quartile-based regression analyses were considered exploratory.

### 2.11. Statistical Analysis

Statistical analyses were performed using MedCalc version 23.4.0 Statistical Software (MedCalc Software Ltd., Ostend, Belgium). Continuous variables were presented as mean ± SD for approximately normal distributions, or median (interquartile range) otherwise. Categorical variables were presented as counts and percentages.

Between-group comparisons at baseline were performed using an independent samples *t*-test (normal distribution) or Mann–Whitney U test (non-normal distribution). Categorical variables were compared using Chi-square or Fisher’s exact test when expected cell counts were <5.

Associations between FMD and inflammatory biomarkers within the post-COVID group were first explored using univariable linear regression (exploratory). Because cardiometabolic factors were imbalanced between groups and may influence endothelial function, multivariable linear regression models were additionally fitted to assess whether biomarker–FMD associations persisted after adjustment for key clinical covariates (see below).

For AF outcomes (t0 and t1), multivariable logistic regression models were specified with group (post-COVID vs. control) as the primary predictor and the following covariates: age, sex, hypertension, obesity (BMI ≥ 30 kg/m^2^), current smoking, dyslipidemia, and HFpEF (and TC/HDL-C where available). The results were reported as odds ratios (ORs) with 95% confidence intervals (CIs). All tests were two-sided, and *p* < 0.05 was considered statistically significant. All regression analyses were considered exploratory and hypothesis-generating.

## 3. Results

### 3.1. Baseline Characteristics

**Figure 1:**
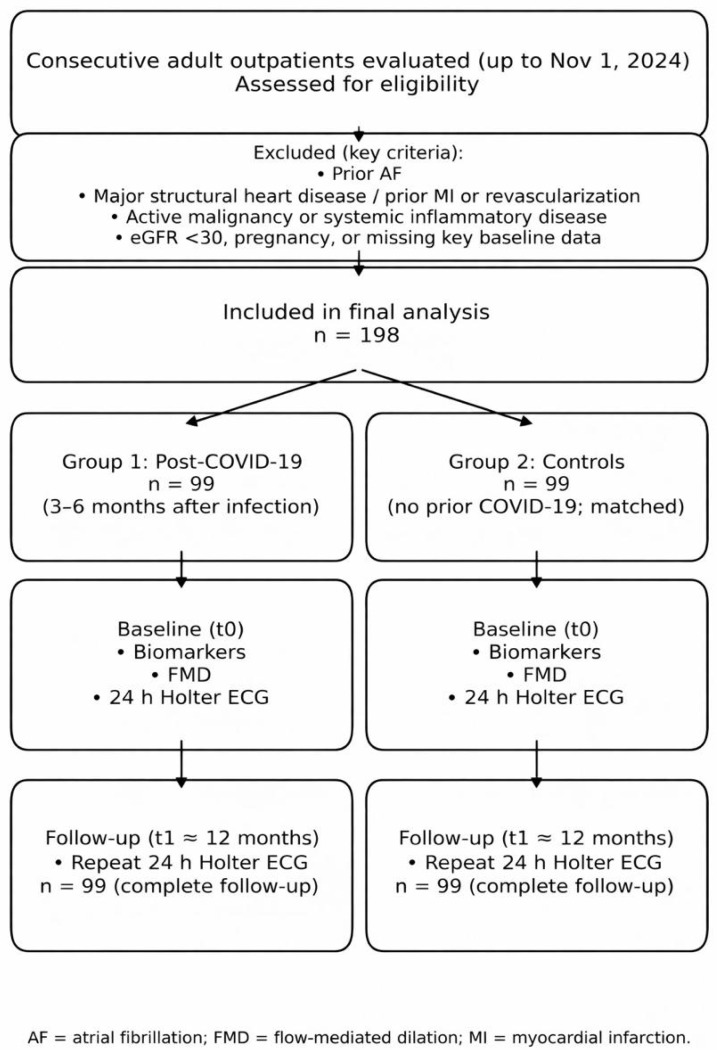
Methods flowchart.

The baseline group characteristics are presented in Figure 1. The two groups were comparable in terms of sex distribution: women represented 53.5% in the post-COVID group (Group 1) and 49.5% in the control group (Group 2), with no statistically significant difference (*p* = 0.5705). The prevalence of hypertension was also similar between groups (51.5% vs. 49.5%; *p* = 0.7767). Given the unequal distribution of key cardiometabolic factors (obesity and current smoking more prevalent in controls; dyslipidemia and HFpEF more prevalent in the post-COVID group), all between-group comparisons were interpreted cautiously. To mitigate confounding, adjusted models including these covariates were prespecified for AF outcomes, and sensitivity analyses were considered (see Statistical Analysis section).

**Figure 2:**
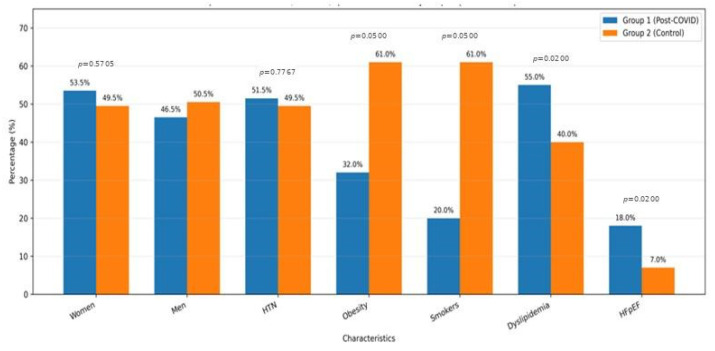
Baseline group characterization according to sex, hypertension (HTN), obesity, current smoking, dyslipidemia, and HFpEF.

In contrast, obesity (Figure 2) was more frequent in the control group (61.0%) compared with the post-COVID group (32.0%), showing borderline statistical significance (*p* = 0.0500). Current smoking was also more common in controls (61.0% vs. 20.0%), likewise borderline (*p* = 0.0500). Dyslipidemia was significantly more prevalent in the post-COVID group (55.0% vs. 40.0%; *p* = 0.0200). Finally, HFpEF was more frequently documented in the post-COVID group (18.0% vs. 7.0%; *p* = 0.0200). These baseline differences indicate an unequal distribution of cardiometabolic risk factors between groups and were addressed in adjusted analyses, as described in the Statistical Analysis section.

### 3.2. Laboratory Parameters and Endothelial Function at Baseline

The mean values (with 95% confidence intervals) of the analyzed parameters are reported in Table 1. Compared with the controls, the post-COVID group showed significantly higher levels of inflammatory and prothrombotic markers, including ESR (*p* < 0.0001), fibrinogen (*p* < 0.0001), CRP (*p* < 0.0001), and D-dimer (*p* < 0.0001).

Regarding hematological indices, the total leukocyte and granulocyte counts were lower in the post-COVID group than in controls (leukocytes: 5.97 vs. 7.72, *p* = 0.0015; granulocytes: 4.12 vs. 5.30, *p* = 0.0017), while the neutrophil-to-lymphocyte ratio (NLR) was higher in the post-COVID group (*p* = 0.0038). Platelet count, hemoglobin, and hematocrit did not differ significantly between groups.

Endothelial function, assessed by FMD, was markedly impaired in the post-COVID group, with significantly lower FMD values compared with controls (7.72 vs. 13.72; *p* < 0.0001).

For lipid parameters, total cholesterol was higher in the post-COVID group (*p* = 0.0154), and the TC/HDL-C ratio was also higher (*p* = 0.0068), whereas HDL-C did not differ significantly between groups.

In the post-COVID group (Group 1), univariable linear regression analyses showed statistically significant inverse associations between FMD and all evaluated predictors (Table 2; all *p* < 0.0001). The strongest association was observed for ESR (R^2^ = 0.6297), indicating that ESR alone explained approximately 63% of the variability in FMD. Fibrinogen also showed a substantial relationship with endothelial function (R^2^ = 0.4855).

Markers reflecting pulmonary involvement and systemic inflammation were likewise associated with reduced FMD, including PGGI (R^2^ = 0.3800), CRP (R^2^ = 0.4210), D-dimer (R^2^ = 0.3333), IL-6 (R^2^ = 0.3416), and ferritin (R^2^ = 0.2742). Hematological indices related to innate immune activation (leukocytes, granulocytes, and NLR) were also inversely associated with FMD.

Because each predictor was assessed in a separate model, these findings should be interpreted as exploratory and do not establish independent effects; multivariable models are required to determine whether these associations persist after adjustment for clinical covariates.

### 3.3. Baseline Atrial Fibrillation (t0)

At baseline (t0), atrial fibrillation prevalence assessed by 24 h Holter ECG was numerically higher in the post-COVID group compared with the controls (25/99 [25.3%] vs. 18/99 [18.2%]); however, this difference did not reach statistical significance (Pearson Chi-square, two-sided *p* = 0.222) (Table 3). These findings indicate that, at study entry, Holter-detected AF prevalence was comparable between groups under the same rhythm monitoring strategy.

### 3.4. Follow-Up Atrial Fibrillation Prevalence (t1)

At 12-month follow-up (t1), atrial fibrillation prevalence assessed by 24 h Holter ECG was higher in the post-COVID group compared with the controls (32/99 [32.3%] vs. 21/99 [21.2%]). This between-group difference reached statistical significance (Pearson Chi-square, two-sided *p* = 0.047) (Table 4).

### 3.5. Quartile Analysis Linking Baseline Inflammation/Endothelial Function to AF at 12 Months (t1)

Consistently, in logistic regression treating NLR quartile as an ordinal predictor, each one-quartile increase in baseline NLR was associated with higher odds of AF at 12 months in both groups: OR per quartile increase 2.55 (95% CI 1.60–4.06; *p* = 0.000082) in the post-COVID group and 2.35 (95% CI 1.39–3.97; *p* = 0.00143) in the controls. In a pooled model, the group × quartile interaction was not significant (p_interaction = 0.82), suggesting comparable NLR quartile slopes between groups.

Participants were stratified into quartiles according to baseline NLR (t0) (Q1 lowest to Q4 highest) (Table 5). Across increasing NLR quartiles, baseline IL-6 increased while baseline FMD decreased (one-way ANOVA across quartiles: IL-6 *p* = 0.003; FMD *p* = 0.002). At 12-month follow-up (t1), AF prevalence increased stepwise across baseline NLR quartiles in both groups, from 8.0% in Q1 to 60.0% in Q4 in the post-COVID group (2/25 vs. 15/25), and from 4.0% in Q1 to 40.0% in Q4 in the controls (1/25 vs. 10/25). The association between baseline NLR quartiles and AF at follow-up was significant in both groups (Pearson Chi-square *p* = 0.0004 for Group 1 and *p* = 0.0078 for Group 2), with a significant dose–response pattern on trend testing (Cochran–Armitage *p* for trend = 0.000022 and 0.00063, respectively).

In multivariable logistic regression including age, sex, hypertension, obesity, current smoking, dyslipidemia, HFpEF, and TC/HDL-C, post-COVID status was not independently associated with AF at baseline (t0). At follow-up (t1), post-COVID status was independently associated with Holter-detected AF. Age, HFpEF, and TC/HDL-C were also independent predictors of AF at t1 (Table 6 and Table 7).

## 4. Discussion

To our knowledge, this is among the first real-world outpatient studies to integrate inflammatory profiling, endothelial function assessment by FMD, and longitudinal Holter-based AF burden in post-COVID patients.

Medical data communicated to date regarding the link between endothelial dysfunction, evaluated using FMD, and COVID-19 infection showed a significant reduction in FMD, but did not identify the link between dysfunction phenotype and arrhythmic endpoint [13]. Additionally, a small prospective post-COVID clinic cohort with FMD evaluation followed for 12 months evaluated vascular recovery but did not provide an arrhythmic subgroup prediction model [14]. This study adds observational evidence that, in a real-world post-COVID outpatient cohort, a persistent inflammatory–prothrombotic profile co-occurs with impaired endothelial function and that AF at one year tends to cluster in participants with higher baseline inflammatory burden. At baseline (t0), the post-COVID group demonstrated substantially higher systemic inflammatory markers (ESR, CRP, fibrinogen) and higher D-dimer levels, together with significantly lower FMD, compared with the controls, consistent with ongoing vascular dysfunction beyond the acute infectious phase. Importantly, these differences were observed despite broadly similar age and sex distribution between groups, suggesting that post-infectious biology may contribute to sustained impairment in endothelial homeostasis [15]. The link between higher levels of coagulation factors and the increasing prevalence of AF has already been mentioned, but without the implication of COVID-19 infection [16].

Beyond serving as a marker of vascular health, endothelial dysfunction may represent a persistent biological substrate linking post-COVID inflammation to long-term cardiovascular vulnerability. Experimental and clinical data indicate that SARS-CoV-2-related endothelial injury can persist well beyond viral clearance, driven by sustained immune activation, oxidative stress, and microvascular remodeling. Reduced nitric oxide bioavailability, increased endothelial permeability, and a shift toward a procoagulant phenotype have all been described in post-acute COVID-19 states, even in clinically recovered individuals. In this context, the markedly lower FMD observed in the post-COVID group may reflect enduring endothelial maladaptation rather than transient post-infectious dysfunction, potentially predisposing to downstream cardiovascular complications, including arrhythmias [1,3,4,6,16].

The regression analyses further support a biologically coherent relationship between inflammation and endothelial dysfunction within the post-COVID cohort. In univariable models, higher levels of ESR, CRP, fibrinogen, D-dimer, IL-6, ferritin, and immune-cell-related indices (including NLR) were each inversely associated with FMD, indicating that greater inflammatory/prothrombotic burden correlated with worse endothelial-dependent vasodilation. While these models are exploratory and do not demonstrate independence, the consistency of direction and strength of associations across multiple pathways (inflammation, coagulation activation, and immune cell activation) reinforces the plausibility of a multi-hit mechanism driving endothelial injury after COVID-19 [13,17,18].

At 12 months (t1), AF occurrence assessed by 24-h Holter monitoring was numerically higher in the post-COVID group compared with the controls, suggesting a trend toward increased atrial arrhythmic burden over follow-up. The quartile analysis complements this observation by demonstrating a graded pattern: higher baseline IL-6 and NLR and lower baseline FMD were associated with a greater clustering of AF cases at one year in both groups. This pattern aligns with the concept that systemic inflammation and endothelial dysfunction are not merely epiphenomena, but may reflect a broader atrial–vascular vulnerability state. Mechanistically, inflammatory signaling can promote atrial electrical instability and structural remodeling through oxidative stress, endothelial activation, microvascular dysfunction, and profibrotic pathways; concomitantly, endothelial dysfunction may impair nitric oxide bioavailability and amplify inflammatory and thrombotic signaling, creating conditions that favor AF initiation and maintenance [19]. Data obtained until now revealed an increased risk of developing atrial arrhythmias in the acute and post-acute phases, but with the attenuation of this risk thereafter [20]. Importantly, the graded increase in AF prevalence across baseline NLR quartiles suggests that these markers may capture a chronic inflammatory tone rather than transient inflammatory activity. NLR integrates innate immune activation and relative lymphocyte suppression and has been linked to adverse cardiovascular outcomes across diverse populations. Similarly, IL-6 plays a central role in sustaining low-grade inflammation, endothelial activation, and prothrombotic signaling. The concordant increase in IL-6 and decline in FMD across NLR quartiles in the present study supports a biologically consistent inflammatory–endothelial axis associated with later AF burden [1,8,12].

Notably, the baseline cardiometabolic profiles were not perfectly balanced between groups: obesity and active smoking were more prevalent in the controls, whereas dyslipidemia and HFpEF were more frequent in the post-COVID group. These imbalances highlight the complexity of interpreting between-group differences and underscore the need for cautious inference. Nevertheless, the robust between-group difference in FMD and the consistent within-group associations between inflammatory markers and FMD support the view that endothelial impairment is meaningfully associated with post-COVID inflammatory burden, beyond traditional risk factor patterns. Clinically, these findings suggest that post-COVID patients with higher inflammatory markers and lower FMD may represent a subgroup warranting closer rhythm surveillance and the more aggressive optimization of modifiable cardiometabolic risk factors.

A growing body of evidence supports the concept of atrial fibrillation as not solely an electrical disorder, but rather the clinical manifestation of complex interactions between inflammation, endothelial dysfunction, and atrial structural remodeling. Endothelial impairment may contribute to AF susceptibility through multiple mechanisms, including altered atrial perfusion, impaired nitric oxide signaling, increased oxidative stress, and the promotion of a local prothrombotic milieu. These processes can facilitate atrial fibrosis and electrical heterogeneity, lowering the threshold for AF initiation and maintenance. The observed association between reduced FMD, elevated inflammatory markers, and subsequent AF burden in our cohort aligns with this atrial–vascular paradigm [2,9,12]. Our findings can also be interpreted within the contemporary concept of atrial cardiomyopathy. Persistent low-grade inflammation and endothelial dysfunction may serve as systemic correlates of a broader atrial remodeling process in which atrial fibrosis, microvascular dysfunction, oxidative stress, and cardiometabolic loading conditions interact to create a pro-arrhythmic and pro-thrombotic milieu. While our study cannot directly phenotype atrial cardiomyopathy due to non-uniform imaging data, the clustering of AF in higher inflammatory strata is compatible with an atrial vulnerability framework rather than a purely electrical disorder [21].

### 4.1. Therapeutic Perspective 

Emerging randomized trial evidence suggests that sodium–glucose co-transporter 2 (SGLT2) inhibitors may reduce incident atrial fibrillation across cardiovascular risk spectra. In a trial-level meta-analysis of 52 randomized controlled trials (112,031 patients), SGLT2 inhibitors were associated with a lower risk of AF overall (RR ≈ 0.86), with subgroup signals suggesting that AF prevention may be more consistent in HFrEF than in HFmrEF/HFpEF populations. Although our study is observational and not designed to test interventions, these data support the hypothesis that endothelial-protective and anti-inflammatory strategies—including SGLT2 inhibition in appropriate clinical contexts (e.g., diabetes and selected HF phenotypes)—could be explored in future prospective trials targeting post-COVID atrial arrhythmic risk [22].

### 4.2. Limitations

Several limitations should be considered. First, the retrospective single-center design is susceptible to selection bias and residual confounding. Second, biomarker–FMD associations were explored using univariable regression and are hypothesis-generating. Without multivariable adjustment for cardiometabolic covariates and considering potential collinearity among biomarkers, independent effects cannot be established and residual confounding is likely. While baseline Holter monitoring was performed systematically, 24-h Holter ECG may underestimate paroxysmal AF burden; therefore, the reported AF prevalence likely represents a conservative estimate of true arrhythmic burden, and longer monitoring would improve sensitivity. Third, the regression models were univariable and therefore exploratory; multivariable modeling is required to clarify whether inflammatory markers, pulmonary CT involvement, and FMD remain associated with AF after adjustment for cardiometabolic covariates (e.g., HFpEF, dyslipidemia, obesity, and smoking). Fourth, the quartile analyses are descriptive and do not provide fully risk-adjusted estimates; therefore, they should be interpreted as hypothesis-generating. Finally, FMD is operator- and protocol-dependent; although measured using a standardized approach, unmeasured procedural variability may affect absolute values. Accordingly, we could not systematically phenotype atrial cardiomyopathy using contemporary imaging markers (e.g., left atrial strain or CMR fibrosis), which may mediate part of the inflammation–AF association.

### 4.3. Implications and Future Directions

Despite these constraints, the study supports a coherent framework linking persistent inflammation, endothelial dysfunction, and AF burden after COVID-19. Prospective multicenter cohorts with standardized endothelial function testing, longer and uniform rhythm monitoring, and comprehensive multivariable adjustment are needed to validate these findings and to define clinically actionable thresholds. If confirmed, an integrated approach combining inflammatory profiling (e.g., IL-6, NLR) with endothelial assessment (FMD) could help stratify post-COVID patients for targeted surveillance and preventive strategies aimed at reducing long-term arrhythmic risk.

## 5. Conclusions

In this single-center observational study, patients evaluated after SARS-CoV-2 infection exhibited a distinctly pro-inflammatory and prothrombotic profile at baseline, accompanied by significantly impaired endothelial function as reflected by lower FMD compared with controls. At 12-month follow-up, atrial fibrillation burden assessed by 24 h Holter monitoring was numerically higher in the post-COVID group, showing a difference in increased AF occurrence.

Across quartiles, higher baseline inflammatory activity (increased IL-6 and NLR) and worse endothelial function (lower FMD) were associated with a greater clustering of AF cases at one year in both post-COVID and control participants, supporting a pathophysiological link between systemic inflammation, endothelial dysfunction, and subsequent atrial arrhythmogenic burden. Together, these findings indicate that persistent inflammatory activation and endothelial impairment may contribute to atrial vulnerability following COVID-19 and may help identify individuals at higher risk for incident or recurrent AF during follow-up.

Given the observational design and baseline differences in cardiometabolic risk factors between groups, the results should be interpreted as associative rather than causal. Larger prospective studies with standardized rhythm monitoring and comprehensive multivariable adjustment are warranted to confirm these relationships and to determine whether targeted anti-inflammatory and endothelial-protective strategies can reduce long-term arrhythmic risk in post-COVID populations.

## Figures and Tables

**Table 1:** Baseline (t0) mean values of analyzed parameters in the post-COVID group (Group 1) and controls (Group 2).

Parameter (Unit)	Group 1 Mean (95% CI)	Group 2 Mean (95% CI)	*p*-Value
Age (years)	63.78 (61.69–66.38)	62.65 (60.29–65.01)	0.5227
ESR (mm/h)	38.06 (32.92–43.19)	16.18 (13.95–18.41)	<0.0001
Fibrinogen (mg/dL)	507.71 (474.83–540.59)	319.83 (283.37–355.33)	<0.0001
CRP (mg/L)	48.40 (36.46–60.34)	7.22 (5.74–8.71)	<0.0001
D-dimer (mg/L FEU)	1.23 (1.04–1.42)	0.55 (0.50–0.60)	<0.0001
Leukocytes (×10^9^/L)	5.97 (5.46–6.47)	7.72 (7.33–8.11)	0.0015
Granulocytes (×10^9^/L)	4.12 (3.65–4.60)	5.30 (5.05–5.54)	0.0017
NLR (unitless)	4.74 (3.70–5.78)	3.14 (2.82–3.45)	0.0038
Platelet count (×10^9^/L)	237.32 (182.05–292.59)	241.53 (215.64–267.42)	0.1860
Hemoglobin (g/dL)	13.10 (12.81–13.40)	13.29 (13.05–13.52)	0.3266
Hematocrit (%)	39.04 (38.19–39.90)	38.75 (38.11–39.40)	0.5883
FMD (%)	7.72 (7.52–7.92)	13.72 (13.35–14.09)	<0.0001
Total cholesterol, TC (mg/dL)	206.02 (198.09–213.95)	193.66 (187.52–199.80)	0.0154
HDL cholesterol, HDL-C (mg/dL)	41.56 (40.27–42.85)	42.04 (40.42–44.27)	0.0647
TC/HDL-C ratio (unitless)	5.18 (4.85–5.51)	4.61 (4.38–4.85)	0.0068

Abbreviations: ESR, erythrocyte sedimentation rate; CRP, C-reactive protein; NLR, neutrophil-to-lymphocyte ratio; FMD, flow-mediated dilation; TC, total cholesterol; HDL-C, high-density lipoprotein cholesterol; FEU, fibrinogen equivalent units.

**Table 2:** Simple linear regression analysis of factors associated with FMD in post-COVID patients (Group 1).

Factor	R^2^	Intercept	95% CI Intercept	Slope	95% CI Slope	*p*-Value
PGGI	0.3800	8.2338	8.0293 to 8.4384	−0.05324	−0.06694 to −0.03953	<0.0001
ESR	0.6297	8.8979	8.6798 to 9.1160	−0.05351	−0.06510 to −0.04191	<0.0001
Fibrinogen	0.4855	9.8691	9.4022 to 10.3360	−0.004219	−0.005094 to −0.003344	<0.0001
CRP	0.4210	8.2509	8.0547 to 8.4472	−0.01082	−0.01333 to −0.008326	<0.0001
LDH	0.1859	8.6063	8.1940 to 9.0186	−0.01429	−0.02075 to −0.007843	<0.0001
D-dimer	0.3333	8.4639	8.1978 to 8.7299	−0.5974	−0.7671 to −0.4277	<0.0001
Ferritin	0.2742	8.2468	8.0058 to 8.4877	−0.002616	−0.004022 to −0.001210	<0.0001
IL-6	0.3416	8.0852	7.8944 to 8.2759	−0.01398	−0.01907 to −0.008887	<0.0001
Leucocytes	0.1306	8.5755	8.0966 to 9.0545	−0.1421	−0.2168 to −0.06728	<0.0001
Granulocytes	0.2397	8.5739	8.2235 to 8.9243	−0.1205	−0.1615 to −0.07962	<0.0001
NLR	0.2304	8.1640	7.9259 to 8.4021	−0.09208	−0.1261 to −0.05827	<0.0001

Univariable (simple) linear regression models assessing associations between baseline variables and flow-mediated dilation (FMD) in the post-COVID group (Group 1). For each model, FMD was the dependent variable. Abbreviations: R^2^, coefficient of determination; CI, confidence interval; ESR, erythrocyte sedimentation rate; CRP, C-reactive protein; LDH, lactate dehydrogenase; IL-6, interleukin-6; NLR, neutrophil-to-lymphocyte ratio; PGGI, pulmonary ground-glass involvement on chest computed tomography (semi-quantitative extent score); FMD, flow-mediated dilation. Note: All tests were two-sided; *p* < 0.05 was considered statistically significant.

**Table 3:** Distribution of patients with atrial fibrillation (AF) at baseline (t0). Atrial fibrillation was assessed by 24 h Holter ECG at baseline (t0) in both groups.

Group	Number of Patients with AF (n)	Percentage (%)	*p*-Value (Chi-Squared)
Group 1 (Post-COVID)	25/99	25.25	0.222
Group 2 (Control)	18/99	18.18	

**Table 4:** Follow-up (t1) atrial fibrillation prevalence on 24 h Holter ECG.

Group	Number of Patients with AF	Percentage (%)	*p*-Value (Pearson Chi-Square, Two-Sided)
Group 1 (Post-COVID)	32	32.32	0.047
Group 2 (Control)	21	21.21	

**Table 5:** Baseline NLR quartiles (t0) and atrial fibrillation at 12 months (t1).

NLR Quartile (t0)	Mean IL-6 (pg/mL)	Mean NLR	Mean FMD (%)	AF at t1—Group 1 (Post-COVID), n/N (%)	AF at t1—Group 2 (Control), n/N (%)
Q1 (lowest)	1.2	1.8	12.5	2/25 (8.0%)	1/25 (4.0%)
Q2	3.5	3.2	10.8	5/25 (20.0%)	3/25 (12.0%)
Q3	7.8	5.6	8.3	10/24 (41.7%)	7/24 (29.2%)
Q4 (highest)	15.6	9.1	6.2	15/25 (60.0%)	10/25 (40.0%)

ANOVA *p*-value across quartiles: IL-6 = 0.003; NLR = 0.003; FMD = 0.002; AF association across quartiles (overall Chi-square): Group 1 *p* = 0.0004; Group 2 *p* = 0.0078; *p* for trend (Cochran–Armitage): Group 1 *p* = 0.000022; Group 2 *p* = 0.00063. Abbreviations: AF, atrial fibrillation; FMD, flow-mediated dilation; IL-6, interleukin-6; NLR, neutrophil-to-lymphocyte ratio. Notes: Quartiles (Q1–Q4) were defined based on baseline NLR (t0) (Q1 = lowest NLR, Q4 = highest NLR). AF at follow-up (t1) was detected by 24 h Holter ECG.

**Table 6:** Multivariable logistic regression for AF outcomes at t0.

Predictor	Adjusted OR (95% CI)	*p*-Value
Post-COVID vs. control	1.33 (0.66–2.69)	0.42
Age (per 1 year)	1.03 (1.00–1.06)	0.058
Female sex	0.92 (0.48–1.74)	0.79
Hypertension	1.41 (0.73–2.73)	0.31
Obesity (BMI ≥ 30)	1.09 (0.56–2.12)	0.80
Current smoking	1.18 (0.61–2.30)	0.62
Dyslipidemia	1.52 (0.81–2.87)	0.19
HFpEF	2.06 (0.93–4.57)	0.074
TC/HDL-C (per 1 unit)	1.20 (0.97–1.49)	0.095

**Table 7:** Multivariable logistic regression for AF outcomes at t1.

Predictor	Adjusted OR (95% CI)	*p*-Value
Post-COVID vs. control	1.88 (1.01–3.52)	0.047
Age (per 1 year)	1.04 (1.01–1.07)	0.012
Female sex	0.95 (0.52–1.74)	0.87
Hypertension	1.36 (0.74–2.51)	0.32
Obesity (BMI ≥ 30)	1.12 (0.61–2.07)	0.71
Current smoking	1.21 (0.66–2.24)	0.54
Dyslipidemia	1.58 (0.89–2.83)	0.12
HFpEF	2.40 (1.12–5.14)	0.024
TC/HDL-C (per 1 unit)	1.24 (1.02–1.52)	0.033

## Data Availability

The original contributions presented in this study are included in the article. Further inquiries can be directed to the corresponding authors.

## Abbreviations

AF	Atrial fibrillation
ACE2	Angiotensin-converting enzyme 2
ANOVA	Analysis of variance
BMI	Body mass index
CI	Confidence interval
CRP	C-reactive protein
CT	Computed tomography
COVID-19	Coronavirus disease 2019
Df	Post-deflation arterial diameter
Di	Baseline arterial diameter
D-dimer	D-dimer fibrin degradation product
ECG	Electrocardiogram
ESR	Erythrocyte sedimentation rate
FEU	Fibrinogen equivalent units
FMD	Flow-mediated dilation
HDL-C	High-density lipoprotein cholesterol
HFpEF	Heart failure with preserved ejection fraction
IL-6	Interleukin-6
LDH	Lactate dehydrogenase
NLR	Neutrophil-to-lymphocyte ratio
NO	Nitric oxide
OR	Odds ratio
PGGI	Pulmonary ground-glass involvement
SARS-CoV-2	Severe acute respiratory syndrome coronavirus 2
SD	Standard deviation
TC	Total cholesterol
TC/HDL-C	Total cholesterol to high-density lipoprotein cholesterol ratio
